# Temporal and geographical variations of chest x-rays: A ten-year register study from Norway

**DOI:** 10.1177/20584601251404163

**Published:** 2025-12-02

**Authors:** Bjørn Hofmann, Ingrid Øfsti Brandsæter, Jan Porthun, Elin Kjelle

**Affiliations:** 1 Centre of Medical Ethics, University of Oslo, Oslo, Norway; 2 Institute for the Health Sciences, 8018 Norwegian University of Science and Technology (NTNU), Gjøvik, Norway; 3 Department of Optometry, Radiography, and Lighting Design, University of South-Eastern Norway (USN), Drammen, Norway

**Keywords:** health services research, x-rays, variation, low-value, appropriateness

## Abstract

**Background:**

Although plain chest x-rays (CXRs) have become standard examinations in many countries, they vary greatly and are recognized as potentially inappropriate imaging procedures.

**Purpose:**

To enhance the safety, quality, effectiveness, and efficiency of healthcare services, by providing knowledge of the temporal and geographical variations in the use of CXRs.

**Materials and methods:**

Outpatient and inpatient data for CXRs was collected for Norway for the years 2013–2022. Data included patients’ age and sex, NCRP code, examination name, modality, hospital/imaging centre, and whether they were in- or outpatients.

**Results:**

On average 648,005 CXRs were performed per year in Norway. This amounts to 123 CXRs per 1000 persons per year (2022). 92% of the examinations were performed at public hospitals, and 39% were outpatient examinations. There was a 17% reduction in CXRs from 2013 to 2022. More male than female patients (54%) got a CXR, especially for the age years 60–79. Geographical variations with a factor of 3.7 and 4.7 were observed for inpatient and outpatient examinations, respectively. The differences between catchment areas decreased from 2013 to 2022.

**Conclusions:**

This is the first study of the number of CXRs from a whole nation for as long as 10 years. It documents substantial geographical variations in number of examinations and a temporal reduction in the total number of examinations. Information of the temporal and geographical variations is crucial for addressing the issue of appropriate imaging and to increase the safety, quality, effectiveness, and efficiency of the healthcare services.

## Introduction

Plain chest x-ray (CXR) is one of the most common examinations in the world and has been crucial for the diagnosis of a range of diseases, such as tuberculosis. CXRs have become routine examinations in many countries, especially for admission, emergency room presentation, ICU, and pre- and postoperative care. While these examinations certainly can be clinically crucial, their extensive use and utility have been questioned.^1–40^ In particular, CXR has been identified as a potential low-value examination.^41^ A recent scoping review identified eighteen studies documenting no altered management after follow-up CXRs in asymptomatic patients performed after procedures known to cause pneumothorax.^42^ Furthermore, routine CXRs have been shown to be ineffective in changing patient management across various scenarios, including preoperative and postoperative screening, hospital admissions, medical checkups, and the staging of cervical and breast cancer. Additionally, repeat CXRs for trauma and ICU patients were found to be of low value.^42^

As for many other health services, geographical variations have been documented for imaging in general^43–46^ and for CXRs in particular.^47^ A study from the USA documented a 1.7 times difference of CXR between Dallas and Seattle (in 2007),^44^ and a study from Switzerland demonstrated how preoperative chest radiography varied from 2.5% to 44.4% across regions (covering 15% of the population from 2013 to 2015).^47^

While geographical variations can reflect demographical differences, unwarranted variations indicate underuse and overuse and challenge the safety, quality, effectiveness, and efficiency of healthcare services.^48^ Moreover, unwarranted variations breach with ethical principles, such non-maleficence, beneficence, autonomy, and justice.^49^ Hence, such variation is an indicator of the goodness of care and can direct our efforts towards effective improvement of the healthcare services.

Accordingly, it is crucial to provide updated knowledge of variations in health services that potentially are unwarranted. The objective of this study therefore is to analyse the temporal and geographical variation in the number of CXR in Norway from 2013 to 2022.

To our knowledge, this is the first study of geographical variations in CXR for a whole country.

## Materials and methods

### Context

**Fig 1.**
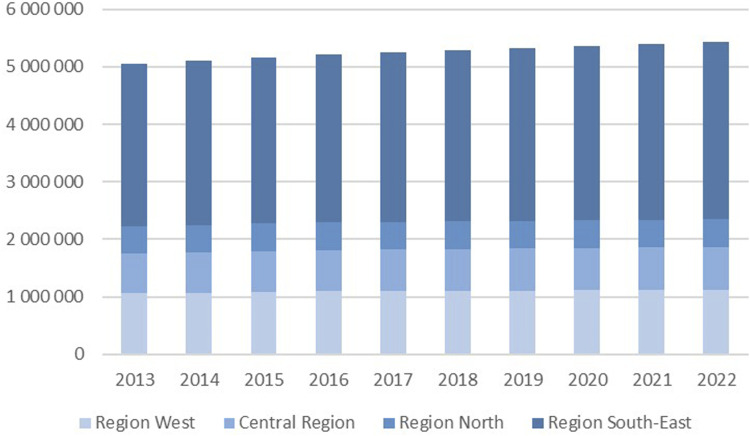
Development in population in each RHA from 2013 to 2022.

Imaging services is a part of the specialist healthcare system in Norway, that is organized in four regional health authorities (RHAs): North, West, Central, and South-East. There are several hospital trusts (HTs) including one or more hospitals within each RHA.^50^ The Region South-East covers about 57% of the Norwegian population as it includes the capital of Oslo and hosts two hospitals with national tertiary specialist health service, such as for oncology, paediatrics, trauma, and transplantations. Fig. 1 shows the population distribution between the RHAs and its development over time.

Imaging is provided by 19 public HTs with one or more imaging departments and by 28 private imaging centres. The private centres are partly commissioned by the RHAs and partly provide outpatient services for private health insurance policies and out-of-pocket payment.^51^ Moreover, some image providers are part of hospitals run by ideal organizations. Some private imaging providers also offer CXR at fee-for-service. These are few and not included.

Norway has a tax-based universal health coverage system with minor co-payments for outpatient services.^51^ The co-payment rate for radiology was €25 per examination in 2023 and has a ceiling of €271 for all services beyond which no co-payment is needed for the rest of the calendar year.^52^

Norway is a very homogenous country with small differences in morbidity and mortality as well as socioeconomic status (https://www.ssb.no/en).^53^

### Materials

The Norwegian Health Economics Administration (HELFO) provided outpatient data, and inpatient data was procured directly from the HTs. Collected data include examination codes for CXR (SC0AA and SSC0AA) in the Norwegian Classification of Radiological Procedures (NCRP) system, name of procedure, modality, hospital/imaging centre, patients’ age and sex, and in- or outpatient status.

Statistics Norway provided data on the Norwegian population in the various geographical areas was provided by for each year and age group. As it takes time for the providers to ascertain the data, and to request, receive, and standardize the data for analyses, the newest data that can be presented is from 2022.

### Methods

Statistical analyses were performed with SPSS Statistics, version 28 (IBM Corp.), and Microsoft Excel 2016 was used for descriptive statistics.

Inpatient data needed to be collected from each HT, making the data extraction process resource-consuming for the HTs. To address this challenge, a representative sample of HTs was selected, allowing for extrapolations that were consistent with population characteristics and outpatient examination data for 32% of the inpatient population. HTs with similar characteristics were paired to minimize potential bias. RHAs and HTs were designated as geographical units in accordance with the organizational structure established by Norwegian health authorities.^54^ Ethical approval was obtained from Regional Committees for Medical and Health Research Ethics (reference 175370) according to Norwegian regulation.

## Results

**Fig 2.**
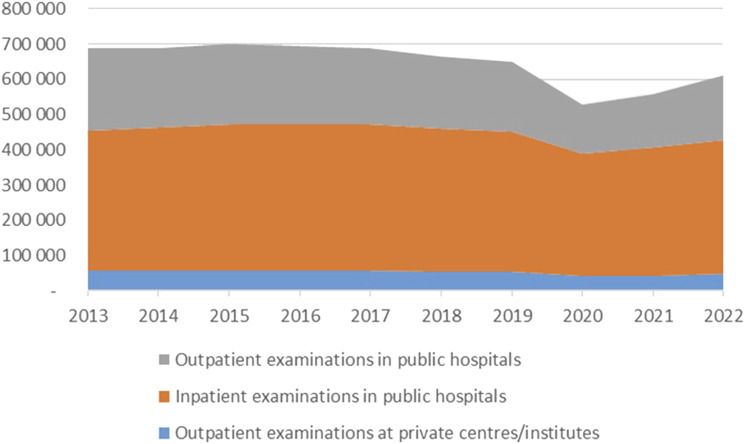
Temporal variations in CXR for the years 2013–2022 for public (in and outpatient) and private outpatient examinations.

On average 648,005 CXRs were performed per year in Norway of which 92% were performed at public hospitals and 39% were outpatient examinations. Fig. 2 shows the temporal development from 2013 to 2022 for in- and outpatient examinations.

The reduction in 2020 was due to the COVID-19 pandemic. In total, there was a 17% reduction in the total number of CXRs from 2013 to 2022. The bulk of this was in outpatient services, amounting to 23% and 35% for private and public providers, respectively.

**Fig 3.**
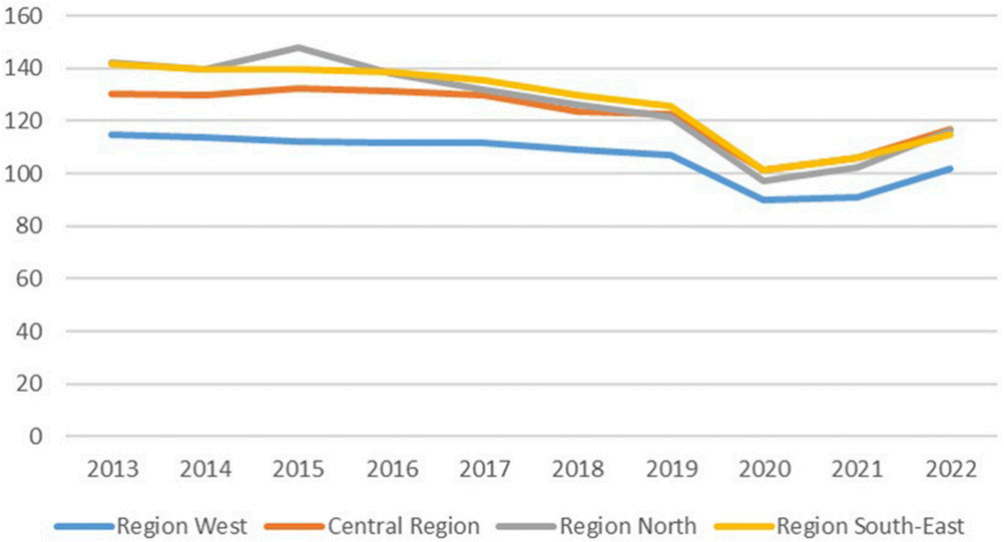
Variation in number of CXR per 1000 inhabitants from 2013 to 2022 for the four health regions.

The average of number of CXRs per 1000 persons per year varied between 133 in 2015 to 94 in 2020. The average for the years from 2013 to 2022 was 123 CXRs per 1000 persons per year, and there was an 18% reduction from 2013 to 2022. Fig. 3 shows the variation in number of CXR per 1000 inhabitants from 2013 to 2022 for the four health regions. On average, Region South-East has taken 16% more CXRs than Region West per 1000 inhabitants per year over the years 2013–2022. The biggest regional difference was in 2015 when Region South-East had 32% more CXRs than the Central Region per 1000 inhabitants. The difference between the regions was reduced from 2013 to 2022, as can be seen in Fig. 3.

**Fig 4.**
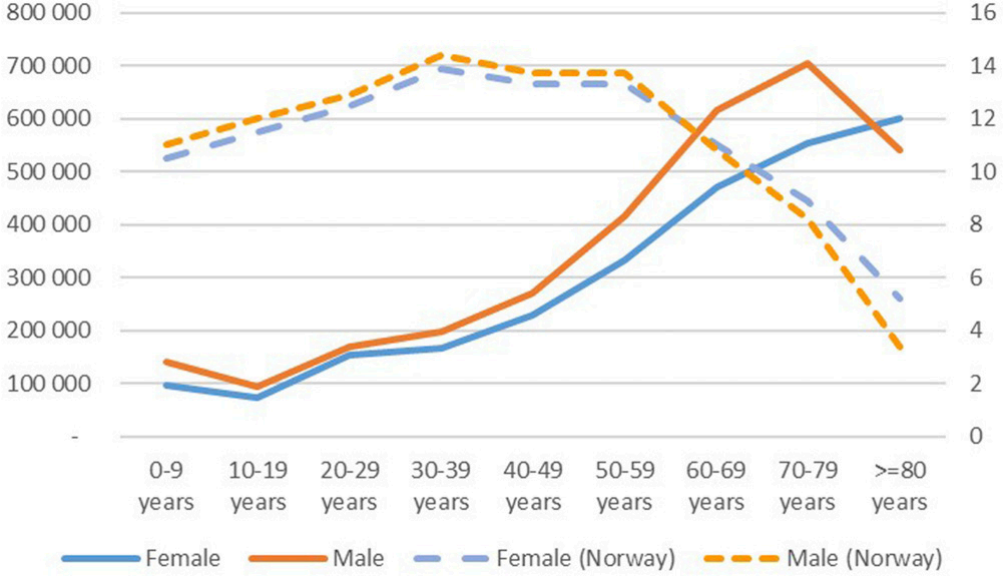
Age distribution for the average number of CXR per year for individuals registered as female and male and the age distribution (dotted lines) for Norwegian population in per cent for 2022.

More male (54%) than female patients got CXRs, especially for the age years 60–79. Fig. 4 shows the distribution for age and registered sex compared to the distribution for the Norwegian population.

**Fig 5.**
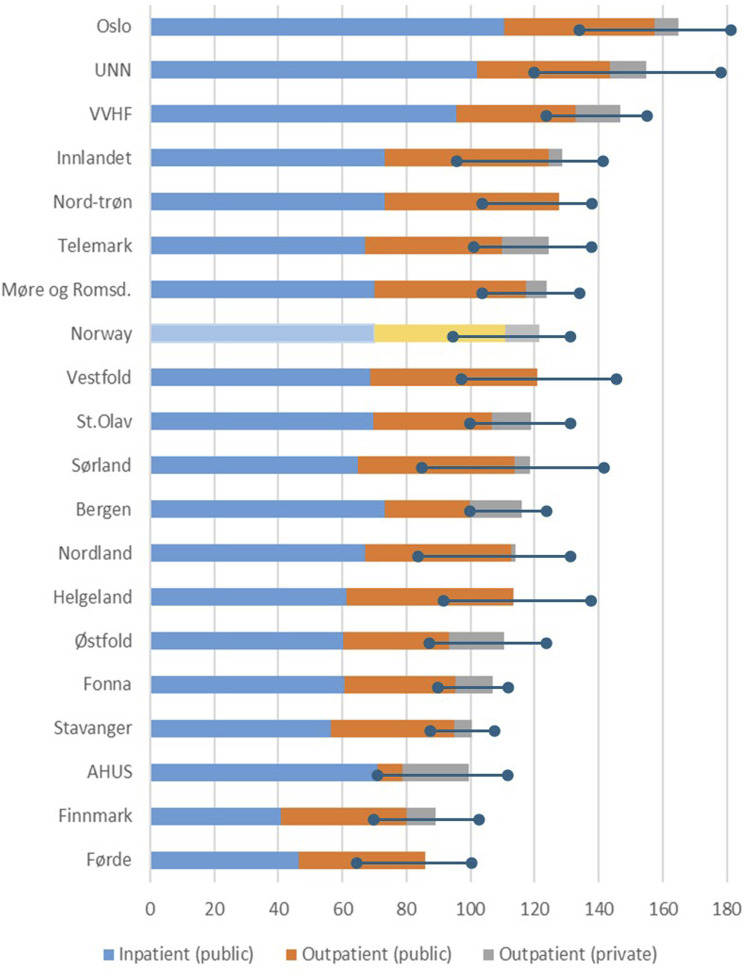
Variation in average number of CXR per 1000 inhabitants from 2013 to 2022 for the catchment areas for public hospitals (inpatient and outpatient) and private outpatient imaging with the variation (maximum and minimum) for all years.

During the years from 2013 to 2022, the maximum difference between catchment areas was 4.0 times more CXRs per 1000 inhabitants per year in areas with the highest compared to the lowest number of examinations. Fig. 5 shows the variation between the catchment areas for public hospitals (inpatient and outpatient) and private outpatient imaging for the average of the years 2013 to 2022.

The number of examinations varied for the local HTs’ catchment areas with a factor of 3.7 and 4.7 (highest to lowest) for inpatient and outpatient examinations, respectively. The differences between catchment areas decreased with about 20% for inpatient and outpatient services from 2013 to 2022.

**Fig 6.**
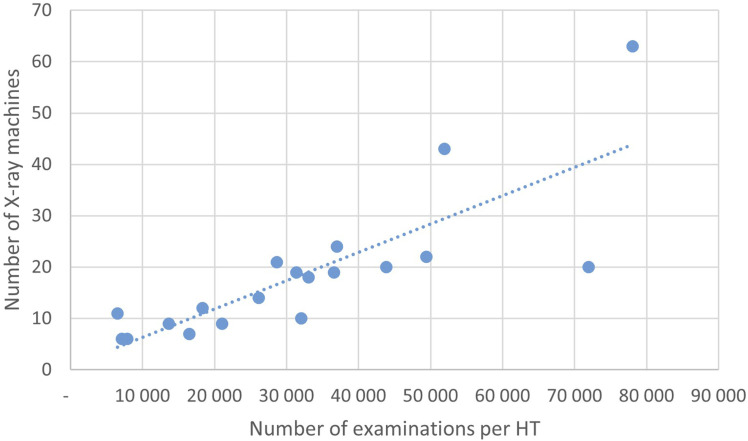
Scatter plot of number of x-ray machines available for CXR per number of CXRs in each HT for 2022.

The number of CXR facilities and radiographer staffing is homogenous throughout the country. The distribution of CXR equipment is also homogenous, and the machine utilization rates can be seen in Fig. 6. The correlation coefficient is 0.81. Two HTs have markedly less examinations than the trendline (Oslo and Bergen), and one HT has more examinations than the trendline (VVHF).

## Discussion

This study shows that a substantial amount of CXRs are taken in Norway each year and that the total number of examinations is decreasing. The decrease started before the COVID-19 pandemic and appears to continue after the pandemic. As expected, there were a reduced number of examinations during the pandemic, but there are no backlog effects found.

While there was a total reduction in CXRs of 17%, the total reduction of imaging in Norway was about 11% and the reduction in conventional radiographs was 9% and when adjusted for the increase in population it was 16%.^55^ During the same period, there was an increase in the number of CTs and MRIs of about 20%.

There is a substantial difference in number of CXRs per 1000 inhabitants taken between the health regions. While the other regions are fairly similar, Region South-East consistently takes more than three times as many CXRs as the other regions. Part of this difference can be explained that this region has some national functions (e.g. for diagnostics and treatments of special diseases), that may require more examinations, but this cannot explain the whole difference.

There are substantial differences in the number of CXRs per 1000 inhabitants between the HTs which cannot be explained by demographic differences. Norway is a homogenous country with small differences in socioeconomic status, morbidity, and mortality. No patterns are found in terms of access to facilities, healthcare personnel, or urbanity. Moreover, there is no association with the total number of inpatient stays for the various HTs either. In particular, there is a strong correlation between the number of x-ray machines and the number of examinations, so the geographical variations cannot be explained by variations in equipment either. Hence, we cannot rule out that the geographical variations are due to differences in appropriateness of imaging and in supply sensitive issues more than in necessary and preference sensitive care.

Our results are in line with findings from the USA^44^ and Switzerland.^47^ While the Swiss study only included 15% of the population, our study accounted for the population in a whole nation. Our results are also in line with other studies of temporal and geographical variations in imaging internationally^44,56–62^ as well as in Norway.^60–66^ They are also consistent with OECD statistics on imaging (https://stats.oecd.org/).

Several studies have documented overuse of CXRs and CXRs being of low value. A recent scoping review of low-value imaging identified 38 studies reporting low-value CXRs.^42^ Of these, 18 studies reported that CXRs did not change management in patients without symptoms in follow-up after procedures known to cause pneumothorax.^2–20^ Routine CXR did not change patient management and was found to be low value in cases such as before and after surgery, at hospital admission, medical check-ups, and in staging of cervical and breast cancer.

Moreover, repeat CXRs in ICU and trauma patients was identified as low-value imaging.^21–40^ A recent study showed that only 65.7% of performed CXRs did not meet American College of Radiology’s Appropriateness Criteria.^67^ Studies also have shown that about one-third of CXRs at hospital admission did not affect diagnosis or treatment^68^ and that there is an overuse of preoperative CXRs.^1^

While the differences between the HTs have been declining from 2013 to 2022, they are still substantial. As pointed out, unwarranted variations in imaging indicate underuse and overuse of health services and challenge the quality, safety, effectiveness, and efficiency of care as well as basic ethical issues. However, it also indicates where we can improve the services. While the variations themselves do not indicate what is ‘right care’ if urges a professional debate on appropriate care.

There are several limitations in this study. The data set on inpatient data is not complete. However, the extrapolations are coherent with outpatient examinations and paired extrapolations reduce the risk of bias.

We have not done advanced statistical analysis, as we are mainly providing descriptive statistics since we analysed data for an entire population. To make results comparable to results from other countries, we have presented them relative to the population. As can be seen from Fig. 4, there are only small differences in the age distribution for the population, while there is a substantial difference in examinations.

The temporal development must be interpreted against demographical changes. In total there is a small increase in the population from 2013 to 2022 (8.3%), and there are only minor changes in the age groups over the study years with some exceptions. There are about 6% fewer younger than 10 years for both female and male, and there are more persons in their 70s (25% and 30% for females and males, respectively) and more persons above 100 (33% and 31%, respectively), but these are few (1082 and 229 for female and male, respectively).

There have been no major economic factors, such as reimbursement changes, guideline implementations, or restrictions in the number of CXRs to be made during a single admission, for example, from Public Health Administrations during the study period. While there has been a Norwegian version of Choosing Wisely Initiative during the study period, it has not targeted CXR as there has been some professional resistance to including these examinations in the initiative. Hence, these factors are unlikely to have any major impact on the temporal developments (or geographical differences).

Moreover, our results are for a specific examination in a particular country for a given period of time. The results cannot be generalized to other health systems. Nonetheless, the results are of interest to other countries for comparison. As the population is very homogeneous in Norway, our results cannot rule out unwarranted variations.

In conclusion, this is the first study of the number of CXRs from a whole nation for as long as 10 years. It documents that almost 650,000 CXRs are performed in Norway per year, amounting to 123 CXRs per 1000 persons per year. 92% of the examinations were performed at public hospitals and more male than female patients (54%) got a CXR. The number of images varied for the local HTs’ catchment areas with a factor of 3.7 and 4.7 (highest to lowest) for inpatient and outpatient examinations respectively. There was a 17% reduction in CXRs from 2013 to 2022, and the differences between catchment areas decreased during this period.

Information of the temporal and geographical variations is a necessary, but not sufficient, condition for addressing the issue of low-value imaging and to increase the safety, quality, effectiveness, and efficiency of the healthcare services.

## Data Availability

Aggregated data without identifyable information are available on relevant request.
